# Advances in Diagnosis and Therapeutic Guidance for Acute Coronary Syndrome: A Narrative of A Precision Medicine Approach

**DOI:** 10.31083/RCM39947

**Published:** 2026-04-14

**Authors:** Takuma Koike, Tomiharu Niida, Seiji Koga, Kenji Yaginuma, Kikuo Isoda, Kenji Inoue

**Affiliations:** ^1^Department of Cardiovascular Biology and Medicine, Juntendo University Nerima Hospital, 177-8521 Tokyo, Japan; ^2^Tokyo Heart Rhythm Clinic Shinjuku, 151-0053 Tokyo, Japan; ^3^Department of Cardiovascular Biology and Medicine, Juntendo University Graduate School of Medicine, 113-8421 Tokyo, Japan

**Keywords:** universal definition of myocardial infarction, dual antiplatelet therapy, high-sensitivity troponin, revascularization strategy, risk stratification, de-escalation therapy

## Abstract

Acute coronary syndrome (ACS) remains one of the leading causes of cardiovascular morbidity and mortality. Recent advances in high-sensitivity cardiac troponin assays, multimodal imaging, and antithrombotic therapies have introduced more individualized risk assessment and management. This review highlights a precision-oriented diagnostic pathway, which integrates the 0/1-hour algorithm with imaging modalities for the comprehensive evaluation of culprit and non-culprit lesions. Revascularization strategies are advancing, with imaging-guided percutaneous coronary intervention (PCI) and coronary artery bypass grafting (CABG), enabling more tailored approaches for complex diseases. Meanwhile, mechanical circulatory support offers potential benefits for cardiogenic shock; nevertheless, there are uncertainties regarding optimal timing and patient selection. Long-term antithrombotic therapy is becoming increasingly optimized. Recent strategies have focused on reducing the duration of dual antiplatelet therapy (DAPT) and exploring de-escalation approaches such as P2Y_12_ inhibitor monotherapy. Secondary prevention extends beyond lipid-lowering therapies and includes metabolic and anti-inflammatory interventions. Collectively, these advancements indicate a shift toward truly personalized care for ACS, aiming to improve outcomes by transitioning away from a one-size-fits-all approach.

## 1. Introduction and Clinical Presentation

### 1.1 Introduction

Acute coronary syndrome (ACS) is characterized by acute myocardial ischemia, which is commonly due to the disruption of vulnerable atherosclerotic plaques and subsequent formation of a thrombus [1]. This disruption leads to a sudden decrease in blood flow to the coronary arteries, resulting in myocardial injury or infarction, depending on the extent and duration of ischemia. ACS comprises three categories: ST-elevation myocardial infarction (STEMI), non-ST-elevation myocardial infarction (NSTEMI), and unstable angina (UA). There are several risk factors for atherosclerotic disease, and by extension, ACS. These risk factors include advanced age, male sex, smoking, hypertension, dyslipidemia, diabetes mellitus (DM), chronic kidney disease (CKD), and family history of premature coronary artery disease. Systemic inflammation, endothelial dysfunction, and prothrombotic state also contribute to plaque vulnerability and risk of rupture. This review offers a concise yet comprehensive overview of contemporary strategies for diagnosing and managing ACS, with emphasis on individualized, precision-based approaches.

This review is organized around five core themes: (1) early strategies for ruling in/ruling out ACS using high-sensitivity troponin, (2) imaging modalities for assessing culprit and non-culprit lesions, (3) decision-making regarding invasive versus conservative management options, (4) individualized antithrombotic strategies, including de-escalation, and (5) secondary prevention strategies guided by cardiometabolic risk profiling.

### 1.2 Clinical Presentation 

Chest discomfort is the primary symptom of ACS. It is typically described as a feeling of pressure, heaviness, or tightness rather than sharp or localized pain. It accounts for over 5% of emergency department visits and may indicate life-threatening conditions, including myocardial infarction (MI), aortic dissection, or pulmonary embolism [2]. Patients often use specific gestures to show discomfort, including placing their hand over their chest (Levine’s sign), pressing their palm (palm sign), or pointing to a specific area (pointing sign). These gestures occur in up to 80% of patients experiencing cardiac chest pain; nevertheless, their diagnostic accuracy is limited [3]. For instance, the pointing sign is highly specific for non-cardiac pain, so it should be interpreted with caution alongside other clinical findings [4, 5]. Ischemic pain may radiate to the epigastrium, jaw, neck, back, shoulders, or arms and sometimes appear without any chest symptoms. In cases of arrhythmias, such as complete atrioventricular block, symptoms, including dyspnea, fatigue, syncope, or altered consciousness, may occur. Atypical or silent presentations are common in older adults or patients with diabetes, and ACS cannot be ruled out based on the absence of symptoms alone. Clinicians should assess the location of pain and its characteristics, duration, triggers, progression, and associated symptoms. Ischemic pain usually develops within minutes, worsens with exertion, and improves with rest or administration of nitroglycerin. Pain influenced by position, movement, or food intake is more likely to be non-cardiac. Pain that is brief, abrupt at onset, or constant over many hours is less typical of ACS.

A focused history that includes previous ischemic episodes, risk factors, and family history is essential. Recurrent ACS often similar to previous episodes, so asking whether current symptoms resemble earlier symptoms can help in making an accurate diagnosis.

## 2. Diagnosis of Acute Coronary Syndrome

### 2.1 Definition and Diagnostics Concepts

#### 2.1.1 Overview

In patients suspected of having ACS, a 12-lead electrocardiogram (ECG) should be conducted within 10 min of the first medical contact, as recommended by current guidelines [6]. Cardiac troponin levels, preferably measured using a high-sensitivity assay, should be promptly obtained to aid in the early determination of whether to rule in or rule out the diagnosis. The Universal Definition of Myocardial Infarction (UDMI) provides standardized criteria for differentiating MI from myocardial injury, guiding clinical management and research classification.

#### 2.1.2 Myocardial Infarction vs. Myocardial Injury

MI is diagnosed using evidence of myocardial necrosis, defined as a cardiac troponin (cTn) level >99th percentile upper reference limit. This diagnosis occurs in a clinical setting consistent with myocardial ischemia, along with a significant increase or decrease in cTn levels. Conversely, myocardial injury is defined as any cTn elevation above the 99th percentile, with or without a dynamic pattern, in the absence of overt ischemia:

Acute myocardial injury: This involves elevated high-sensitivity cardiac troponin (hs-cTn) levels that show a rise and/or fall but lack evidence of ischemia (symptoms, ECG changes, and imaging). Common causes include heart failure, arrhythmia, sepsis, renal dysfunction, anemia, and respiratory failure.

Chronic myocardial injury: This involves persistent hs-cTn elevation without significant temporal variation, which is typically observed in patients with structural heart disease, CKD, or stable heart failure.

#### 2.1.3 Troponin Dynamics: Rise And Fall

The “rise and/or fall” of troponin levels is essential in differentiating between acute and chronic injuries. Based on widely accepted criteria:

If the initial hs-cTnT level was <99th percentile, a subsequent increase > of >7 ng/L was deemed significant [7].

If initial hs-cTnT ≥99th percentile, a ≥20% increase is required (18 ng/L → ≥21.6 ng/L) [8].

#### 2.1.4 Type 1 Myocardial Infarction (T1MI)

Type 1 Myocardial Infarction (T1MI) results from atherothrombotic plaque disruption (rupture, erosion, or dissection) resulting in acute coronary occlusion. It requires:

At least one hs-cTn value >99th percentile with rise and/or fall.

Clinical evidence of ischemia (symptoms, ECG changes, imaging, or angiographic findings).

In unclear cases, findings such as dynamic ECG changes or ischemic imaging support the diagnosis. Coronary angiography conducted within 30 days post-discharge in patients experiencing elevated hs-cTn levels but no alternative explanation (anemia, AF, or renal failure) may support the diagnosis of T1MI.

#### 2.1.5 Type 2 Myocardial Infarction (T2MI)

Type 2 Myocardial Infarction (T2MI) is due to a mismatch between the myocardial oxygen supply and demand without acute plaque rupture. It also requires:

Hs-cTn elevation with dynamic change.

Clinical context of supply-demand imbalance (anemia, infection, heart failure, tachyarrhythmias, and hypoxia).

Differentiating between T1MI from T2MI can be difficult without angiographic data. A documented thrombus usually indicates T1MI; however, angiography often lacks sensitivity. In these situations, contextual adjudication is essential, and an “undetermined MI type” category may be used.

#### 2.1.6 Unstable Angina (UA)

UA is defined as myocardial ischemia that occurs without a significant increase or dynamic change in hs-cTn levels to meet the criteria for MI. Troponin levels may either remain within normal limits or >99th percentile, given that they are stable over time. UA should be suspected in patients experiencing ischemic symptoms with one or more of the following conditions:

New-onset angina.

Rest angina lasting ≥20 min.

Crescendo or worsening angina.

Even if hs-cTn levels are elevated or stable, UA may still be diagnosed if ischemic symptoms are present and angiography shows ≥75% stenosis. However, if no significant lesions are found, a diagnosis of chronic myocardial injury should be considered.

#### 2.1.7 Differentiating Diagnoses in Troponin-Negative Patients

In patients with normal hs-cTn levels and no rise/fall pattern (ruled out by the 0/1-hour algorithm), further differentiation can be made based on:

Non-cardiac chest pain: no ischemic features and normal ECG/imaging.

Cardiac but non-coronary causes: for instance, pericarditis, myocarditis, Takotsubo syndrome.

Coronary causes (UA): ischemic symptoms and corroborating clinical findings.

The clinical context, symptom characteristics, ECG findings, imaging results, and serial biomarkers must be considered.

### 2.2 Initial Triage and Electrocardiogram Evaluation

For patients suspected of having ACS, acquiring and interpreting a 12-lead ECG within the first 10 min of their presentation is crucial for the initial triage [9]. Based on the ECG findings, patients were provisionally categorized as having STEMI or non-ST-segment elevation ACS (NSTE-ACS). The presence of ST-segment elevation and chest pain strongly indicates STEMI, which necessitates immediate reperfusion therapy. Conversely, patients experiencing chest pain without ST-segment elevation are categorized as having NSTE-ACS, which includes NSTEMI and UA, and is further differentiated by serial cardiac troponin (cTn) measurements. These categories, based on ECG, are preliminary, and the final diagnosis may vary after further evaluation. In early-phase MI, ST elevation may not be apparent. Changes in T-wave morphology, such as hyperacute or tall T-waves, may precede ST elevation [10]. Reciprocal ST depression may also help identify the affected lesions. In cases of inferior MI, right precordial leads (V4R) can help in assessing right ventricular involvement. However, ST elevation in these leads may resolve within 10 h [11]. Patients with bundle branch block or ventricular pacing may experience obscured ischemic changes. A new left bundle branch block (LBBB) indicates extensive ischemia, while a new right bundle branch block indicates septal involvement [12].

### 2.3 Cardiac Biomarkers and the 0 h/1 h Algorithm

**Fig. 1.**
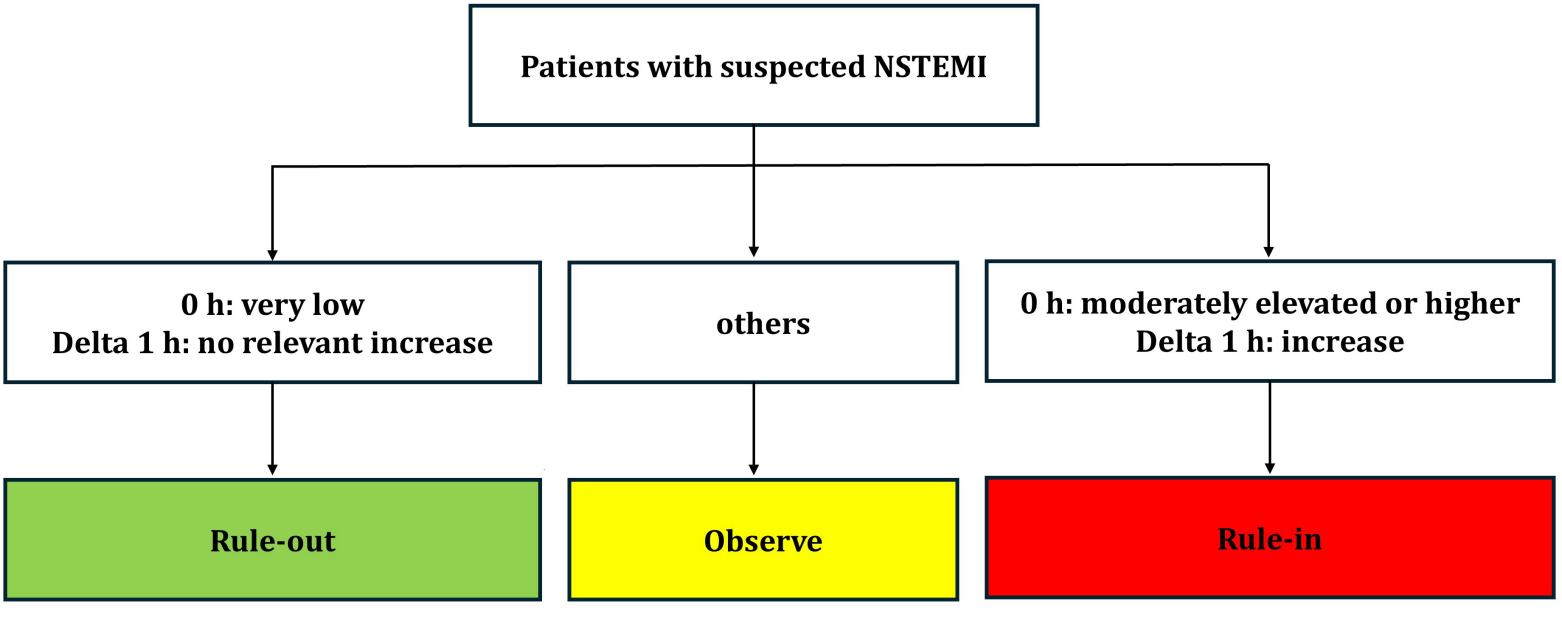
**Flowchart illustrating the 0/1-hour algorithm for the triage of patients with suspected non-ST-elevation myocardial infarction**. Definitions: 0 h = hs-cTn at presentation to the emergency department. Delta 1 h = a bsolute change in hs-cTn within the first hour. NSTEMI, non-ST-segment elevation myocardial infarction; hs-cTn, high-sensitivity cardiac troponin.

HHs-cTn assays enable earlier and more accurate detection of MI. The 0-hour/1-hour algorithm categorizes patients into three diagnostic pathways: rule-out, rule-in, and observation. This categorization was based on the initial hs-cTn levels and their change after 1 h (Fig. 1) [13].

Cutoff values vary by assay.

Rule-out: Very low or unchanged low hs-cTn levels; >99% negative predictive value.

Rule-in: High or significantly increasing hs-cTn; ~70–75% positive predictive value.

Observe: Intermediate hs-cTn values carry similar risks to rule-in and require further testing.

If troponin levels show no significant increase or decrease, and the patient is experiencing worsening chest pain at rest, UA should be considered. Risk stratification tools can help guide further management. Invasive coronary angiography helps differentiate these entities; however, it is not always essential [10]. In cases where there is no rise or fall in troponin level, but the patient presents with worsening chest pain at rest, UA must be considered. Several scoring systems can assist in risk stratification [14, 15, 16, 17]:

Thrombolysis in Myocardial Infarction (TIMI): Based on age, risk factors, aspirin use, recent symptoms, ECG changes, and biomarkers.

Global Registries of Acute Coronary Events (GRACE): Includes age, heart rate, blood pressure, creatinine, Killip class, cardiac arrest, ST deviation, and troponin levels.

HEART: Includes history, ECG, age, risk factors, and troponin.

EDACS: Includes demographics, symptoms, and hs-cTn over 2 h.

This algorithm enhances accuracy, reduces emergency department (ED) stays, and lowers costs. However, these values must be interpreted considering the timing of symptom onset, age, and renal function [18].

### 2.4 Non-Invasive Imaging

Non-invasive imaging is crucial in diagnosing and managing early- or intermediate-risk cases.

Transthoracic echocardiography (TTE) detects wall motion abnormalities and evaluates the left ventricular (LV) function. It can identify complications, including pericardial effusion, ventricular thrombus, and mitral regurgitation.

Coronary CT angiography (CCTA) is useful in low-to-intermediate-risk cases, as it provides a high negative predictive value [19]. The morphology of plaques observed through CCTA correlated with future events. However, this method may increase costs and does not shorten hospitalization time.

Fractional flow reserve derived from computed tomography (FFR-CT) enhances specificity and reduces the need for unnecessary invasive testing [20]. Recent evidence also supports the use of CCTA and FFR-CT in identifying high-risk plaque features and functionally significant non-culprit lesions. This can aid in long-term risk stratification and revascularization planning [21, 22].

When combined with intravascular imaging modalities, such as intravascular ultrasound (IVUS) and optical coherence tomography (OCT), this multimodal approach enables precise characterization of culprit and non-culprit lesions, thereby providing individualized treatment strategies for ACS. The plaque morphology observed through CCTA is associated with future coronary events. Cardiac Magnetic Resonance Imaging (CMR) assesses myocardial viability and infarction patterns, while differentiating ACS mimics, such as Takotsubo syndrome or myocarditis. Stress imaging (scintigraphy, stress ECG) can visualize ischemia but may trigger symptoms or hemodynamic instability, which limits their effectiveness in acute utility.

#### Clinical Implications – Diagnosis of Acute Coronary Syndrome

Early and accurate diagnosis of ACS relies on integration combination of symptom assessment, ECG interpretation, hs-cTn dynamics, and adjunctive imaging. The adoption of the 0/1-hour algorithm has significantly enhanced the efficiency of rule-in and rule-out processes in emergency settings. However, differentiating between MI types, particularly T1MI vs. T2MI, in real-world practice is challenging. Clinicians must be vigilant of atypical presentations, especially in older adults or patients with diabetes, and should interpret hs-cTn changes in the context of comorbidities, timing of symptoms, and renal function. Non-invasive imaging modalities such as echocardiography and CCTA support risk stratification and guide downstream testing or intervention. Ultimately, adopting a multimodal, time-sensitive approach improves diagnostic accuracy and supports individualized care strategies.

## 3. Medical Management and Invasive Strategy

The initial management of patients suspected of having ACS aims to stabilize their symptoms, reduce infarct size, and prevent additional thrombotic complications. In cases of STEMI, timely reperfusion is vital, ideally through primary percutaneous coronary intervention (PCI) within 120 mins of the first medical contact. If timely PCI is not possible, fibrinolytic therapy, followed by transfer to a PCI-capable center, is an acceptable alternative. In NSTE-ACS, early risk assessment determines the urgency of intervention. This may involve immediate angiography in unstable patients or delayed evaluation in low-risk individuals. Ongoing ECG monitoring is crucial during the acute phase to detect any dynamic changes or arrhythmias. Oxygen supplementation is only recommended when the oxygen saturation falls below 90%. Nitrates, either sublingual or intravenous, may alleviate ischemic symptoms unless contraindicated because of hypotension or right ventricular infarction. Opioids can be administered for pain unresponsive to nitrates; nonetheless, their impact on outcomes remains uncertain. Antiplatelet therapy is fundamental in managing ACS. Aspirin should be administered promptly in a loading dose of 150–300 mg unless contraindicated. A P2Y_12_ receptor antagonist such as clopidogrel, prasugrel, or ticagrelor should be added to create dual antiplatelet therapy (DAPT) [23]. In patients with STEMI, early loading with a P2Y_12_ inhibitor before PCI is associated with improved reperfusion efficacy. However, for cases of NSTE-ACS, guidelines recommend deferring P2Y_12_ administration until the coronary anatomy is defined to avoid unnecessary exposure in patients who might need surgical revascularization.

The timing of coronary angiography in NSTE-ACS is based on risk levels:

Very high-risk: Hemodynamic instability, arrhythmias, mechanical complications → immediate angiography.

High-risk: Rule-in by hs-cTn, dynamic ECG changes, GRACE >140 → within 24 h.

Low-to-intermediate risk: Use non-invasive imaging before angiography.

For patients unsuitable for invasive procedures (older adults and the frail), DAPT and secondary prevention remain essential for reducing cardiovascular events [24].

The choice of P2Y_12_ inhibitor depends on bleeding risk, previous medication exposure, and procedural considerations [25]. Prasugrel and ticagrelor provide faster and more potent platelet inhibition than clopidogrel, but they do not pose a higher bleeding risk. In situations where surgery is anticipated or patients are at a high risk of bleeding, clopidogrel remains a practical alternative. Intravenous cangrelor is available, which requires rapid platelet inhibition with a short offset, such as in patients requiring urgent surgery. For patients undergoing PCI, DAPT is recommended for at least 12 months, unless the bleeding risk requires a shorter duration. In patients who have an elevated risk of gastrointestinal bleeding, the co-prescription of proton-pump inhibitors (PPIs) is recommended. For patients requiring long-term oral anticoagulation (atrial fibrillation (AF)), the initial period of triple therapy should be brief, ideally 1 to 4 weeks. After this period, aspirin should be discontinued, and P2Y_12_ inhibitor monotherapy should continue.

### Clinical Implications

The initial management of ACS requires prompt symptom control, risk stratification, and the early initiation of evidence-based pharmacotherapy. For patients with STEMI, timely reperfusion is crucial, ideally through primary PCI within 120 mins, to improve outcomes. For cases of NSTE-ACS, a risk-based strategy guides the timing of coronary angiography, with immediate intervention reserved for patients who are unstable or those at high risk. Antiplatelet therapy is essential; however, the timing and selection of P2Y_12_ inhibitors should account for procedural plans and bleeding risks. Invasive evaluation should be paired with ongoing ECG monitoring and individualized pharmacological support, including careful use of nitrates, oxygen, and analgesics. Overall, personalized decision-making that balances the ischemic urgency against procedural risk is key to optimizing outcomes during the early phase of ACS.

## 4. Revascularization Strategy: PCI and CABG

Revascularization remains essential to treating ACS, with PCI being preferred in most patients, particularly in cases of STEMI. Timely reperfusion correlates strongly with improved outcomes. Drug-eluting stents (DES), especially newer-generation devices, have reduced restenosis and stent thrombosis compared to earlier iterations [26]. Balloon angioplasty alone is rarely used in ACS situations, except in specific types of lesions or resource-limited environments. For patients with complex multivessel disease or left main coronary artery involvement, especially those with high SYNTAX scores, CABG remains the preferred strategy, as recommended by the current clinical guidelines.

### 4.1 Intravascular Imaging

Intravascular imaging, including intravascular ultrasound (IVUS) and optical coherence tomography (OCT), plays a central role in contemporary PCI by providing detailed information on lesion morphology, plaque burden, vessel size, and stent expansion. These modalities enable more precise procedural planning and optimization than angiography alone.

A 2024 meta-analysis demonstrated that intravascular imaging–guided PCI significantly reduced the risk of target lesion failure compared with angiography-guided PCI (risk ratio [RR] 0.71, 95% CI 0.63–0.80; *p* < 0.0001) [27]. When evaluated separately, both IVUS-guided and OCT-guided PCI showed favorable trends; however, statistical significance was achieved only for IVUS (RR 0.66, 95% CI 0.56–0.78; *p* < 0.0001). A direct comparison between OCT and IVUS revealed no significant difference in clinical outcomes (RR 0.89, 95% CI 0.65–1.22; *p* = 0.47).

The importance of intravascular imaging is further magnified in ACS, where culprit lesions are frequently complex and characterized by thrombus-rich, unstable plaque. Imaging-assisted PCI offers several advantages in this setting, including:

Accurate characterization of plaque composition and burden.

Avoidance of procedural complications such as distal embolization.

Optimization of stent diameter, length, and expansion.

Guidance for post-dilation and assessment of residual dissections.

Among available tools, OCT provides the highest spatial resolution (10–20 µm), enabling detailed visualization of microstructures within the vessel wall. This allows precise classification of culprit lesions into distinct pathological morphologies—plaque rupture, plaque erosion, and calcified nodule—each associated with unique clinical and procedural implications.

Overall, the integration of IVUS or OCT into PCI strategy supports individualized treatment and has been consistently associated with improved procedural quality and better clinical outcomes.

#### Clinical Implications 

Plaque rupture, discovered in approximately 40% of ACS cases, is defined as a fibrous cap discontinuity underlying the formation of a cavity. These lesions often present with a heavy thrombus burden, which increases the risk of non-reflow, distal embolism, and tissue protrusion after stenting [28]. OCT aids in assessing the thrombus extent, the need for additional stenting, and guiding long-term therapy. Plaque erosion, observed in younger individuals, females, and smokers, is characterized by an intact fibrous cap with surface irregularities or an attached thrombus. OCT helps in identifying these lesions, which may be managed conservatively or with minimal intervention. This allows individualized antithrombotic strategies and potentially shorter DAPT. Calcified nodules were identified as irregular protruding calcifications with surface disruption and thrombus formation. Common in older adults or patients with CKD, they are technically challenging and related to a higher risk of stent under-expansion. OCT facilitates appropriate device selection for atherectomy, intravascular lithotripsy, and precise stent deployment. This imaging-guided, morphology-based approach, where precision and tailored interventions are emphasized, enables clinicians to deliver highly individualized revascularization therapy based on detailed intravascular pathology, which is relevant, especially in Japan. Thrombus aspiration may reduce no-reflow and embolic risk in lesions with a large thrombus burden; however, it is not routinely recommended because of potential complications, such as embolization to other organs. Selective use is advised in patients with a high thrombus burden. Rotational atherectomy, laser atherectomy, and other debulking techniques may be required to treat heavily calcified and complex lesions [29]. This technique is not routinely used in ACS; however, it is valuable in select cases when conducted by experienced operators. Recently, new concepts of MI with nonobstructive coronary arteries (MINOCA) were proposed. Spontaneous coronary artery dissection (SCAD) is one of the possible causes of MINOCA [30]. SCAD is more common in women under the age of 50. Most SCAD cases can be easily distinguished angiographically from alternative causes of ACS; however, this is sometimes difficult [31]. In such cases, intravascular imaging, especially OCT, is useful for diagnosing SCAD owing to the presence of a false lumen within the coronary artery wall [30]. Conversely, it carries a small additional procedural risk in the fragile arteries with SCAD, particularly in cases of severe tortuosity or when SCAD occurs in distal, small-calibre arteries. PCI for SCAD is associated with high complication rates and lower angiographic success rates, while conservative management is related to complete coronary healing in most cases [31]. Furthermore, the clinical impact of imaging-guided PCI (OCT or IVUS) in SCAD remains unclear. Further studies that determine the optimal diagnostic and treatment strategies for these patients are required.

### 4.2 Coronary Artery Bypass Grafting

Revascularization in multivessel disease needs to be carefully assessed. Approximately 50% of the patients with ACS experience multivessel coronary artery involvement [32]. In hemodynamically stable individuals, complete revascularization either during the index procedure or in a staged manner enhances long-term outcomes. Conversely, in cardiogenic shock, the focus should be on the culprit lesion, as reported by recent randomized trials. CABG is crucial in patients with complex coronary anatomy, left main disease, or mechanical complications, such as ventricular septal rupture or papillary muscle rupture. Delaying surgery for at least 48–72 h after symptom onset may reduce perioperative mortality in stable patients requiring CABG. In hemodynamically stable patients with ACS, evidence so far shows that immediate CABG, especially within the first 24 h, may lead to increased perioperative mortality. Multiple observational studies have shown that delaying surgery for 2–7 days after initial symptoms may reduce in-hospital mortality compared with earlier intervention [33, 34, 35, 36, 37]. Current guidelines recommend approaching a heart team for decision-making in patients with high SYNTAX scores, DM, or equivocal angiographic findings. Risk scoring systems such as SYNTAX and EuroSCORE provide insights into anatomical and surgical complexity, respectively. However, clinical judgment is essential, as frailty, comorbidities, and procedural logistics often affect the choice of treatment. Integration of imaging data, hemodynamic status, and patient preferences is crucial for devising personalized revascularization plans. In patients with multivessel coronary disease who present with STEMI, the optimal strategy for non-culprit lesion revascularization remains an area of active investigation. Angiography- and physiology-guided approaches (Fractional flow reserve (FFR)) have been evaluated to identify functionally significant lesions beyond the culprit vessel. A recent meta-analysis that compared these strategies observed no significant difference in major cardiovascular outcomes but suggested that angiography-guided complete revascularization may provide logistical advantages in certain settings [38]. These findings support a patient-tailored approach that considers clinical stability, anatomical complexity, and institutional expertise when planning multivessel PCI.

#### Clinical Implications

The revascularization strategy in ACS must be tailored to coronary anatomy, hemodynamic stability, and patient-specific factors. Primary PCI is the preferred approach in STEMI, while risk-based timing guides angiography is preferred in NSTE-ACS. Intravascular imaging with IVUS or OCT enhances stent optimization and improves outcomes, especially in complex lesions. Morphology-guided strategies, such as conservative treatment for plaque erosion or preemptive atherectomy for calcified nodules, permit a more nuanced approach. CABG remains an important option for patients with multivessel or left main disease, especially those with diabetes or high SYNTAX scores. In cardiogenic shock, culprit-only revascularization is preferred. Overall, integrating anatomical, physiological, and clinical data supports individualized revascularization strategies that balance completeness, safety, and long-term efficacy.

## 5. Hemodynamic Support in Cardiogenic Shock

### 5.1 Cardiogenic Shock

Cardiogenic shock (CS) is a critical complication of acute myocardial infarction (AMI) that occurs in approximately 5–10% of cases. It is characterized by inadequate tissue perfusion due to a severe reduction in cardiac output, frequently resulting from extensive left ventricular dysfunction. The in-hospital mortality from AMI complicated by CS remains high and often exceeds 40% despite advances in reperfusion therapy. Initial pharmacological management includes vasoactive agents such as norepinephrine, dopamine, and dobutamine, tailored to hemodynamic profiles. However, these agents may increase myocardial oxygen demand and exacerbate ischemia, highlighting the limitations of pharmacological therapy alone. Identifying and escalating mechanical circulatory support (MCS) early is essential in patients with persistent hypotension, signs of end-organ hypoperfusion, or inadequate response to inotropes [39]. Time is a key factor in shock progression. Observational studies report that nearly half of patients develop CS within 6 h of AMI onset and over 70% within 24 h. Therefore, even initially stable patients require close hemodynamic monitoring. Transthoracic echocardiography should be conducted promptly to assess left ventricular function, identify valvular abnormalities, and detect mechanical complications, including ventricular septal rupture or papillary muscle dysfunction. In Table 1, the Society for Cardiovascular Angiography and Interventions (SCAI) proposes a 5-stage classification system (A–E) for cardiogenic shock [40], aiding communication among providers and supporting clinical decision-making. This framework considers hemodynamic parameters, metabolic markers, and clinical trajectories, enabling tailored escalation of care.

**Table 1.** **Severity Classification of Cardiogenic Shock (SCAI) stages of cardiogenic shock**.

Stage	Clinical phenotype	Typical objective signals
**A**	No shock at present; risk context (e.g., large MI/acute HF).	Blood pressure and perfusion maintained; no significant metabolic/organ injury signals.
At risk		
**B**	Early hemodynamic stress without clear hypoperfusion.	Relative hypotension and/or tachycardia; biomarkers may be minimally abnormal.
Beginning CS		
**C**	Clear hypoperfusion requiring vasoactive agents and/or temporary mechanical support.	Lactate elevation and/or evolving organ dysfunction can be present; invasive hemodynamics may show reduced forward flow with elevated filling pressures.
Classic CS		
**D**	Failure to stabilize; worsening despite initial support.	Need for multiple vasoactive agents and/or device escalation to maintain perfusion.
Deteriorating/doom		
**E**	Collapse/arrest physiology with ongoing resuscitation.	Severe acidosis/high lactate and refractory malignant arrhythmias may occur; requires maximal support.
Extremis		

The patients were stratified into five stages (A–E) based on their clinical presentation, bedside findings, laboratory markers, and hemodynamic parameters. This staging system helps to standardize communication, risk stratification, and therapeutic decision-making in the management of cardiogenic shock. CS, cardiogenic shock.

### 5.2. Mechanical Circulatory Support Devices

For patients with refractory cardiogenic shock, MCS devices provide hemodynamic stabilization, preserve end-organ function, and enhance survival. Device selection and initiation timing are important because delayed deployment may limit benefits. The main MCS options include an intra-aortic balloon pump (IABP), microaxial flow pumps (Impella), and venoarterial extracorporeal membrane oxygenation (VA-ECMO).

#### 5.2.1 IABP

IABP improves coronary perfusion and reduces afterload through inflation during diastole and deflation during systole. Historically deemed the first-line treatment for CS, its routine use has declined following the IABP-SHOCK II trial, which showed no significant mortality benefit after 30 days or 12 months in patients with AMI and CS undergoing PCI [41, 42]. Nevertheless, IABP may still be appropriate in specific scenarios such as mechanical complications or as a bridge to more advanced support. The IABP-SHOCK II risk score with variables including age, serum lactate level, renal function, and left ventricular ejection fraction to guide early triage and inform the potential need for escalation to advanced MCS [43].

#### 5.2.2 Impella (Microaxial Flow Pump)

The Impella is a percutaneously implanted microaxial pump that unloads the left ventricle while maintaining systemic perfusion. It draws blood from the left ventricle and delivers it to the ascending aorta, reducing the myocardial oxygen consumption and wall stress. Impella provides more substantial hemodynamic support than IABP. Registry data and small trials indicate that early Impella implantation before PCI may confer survival benefits in specific patients [44]. However, its use is associated with vascular complications, hemolysis, and high costs. Indications included high-risk PCI, severe left ventricular dysfunction, and cases that warranted left ventricular unloading [45]. Notably, the DanGer Shock trial, published after the most recent European Society of Cardiology (ESC) guidelines, was the first randomized controlled trial to show a survival benefit with the early use of a microaxial flow pump, Impella CP (Abiomed, Danvers, MA, USA), and cardiac power in patients with AMI-related cardiogenic shock. This trial showed a significant reduction in all-cause mortality after 180 days compared to standard care, supporting the hemodynamic advantages of actively unloading the left ventricle. These findings led to a class IIa recommendation in the 2025 American College of Cardiology/American Heart Association guidelines for specific patients. The ESC 2023 guidelines predated this publication; nevertheless, future ESC guideline updates should re-evaluate the role of Impella, considering this pivotal evidence. By integrating such trial data into practice guidelines, there is potential for further standardization and justification of early mechanical support strategies for appropriately selected patients.

#### 5.2.3 Veno-Arterial ECMO (VA-ECMO)

VA-ECMO offers full cardiopulmonary support by draining venous blood (typically from the femoral vein), oxygenating it through a membrane oxygenator, and returning it to arterial circulation (typically through the femoral artery). It is most beneficial in cases of profound cardiogenic shock with multi-organ failure, especially when combined cardiac and respiratory support is needed. ECMO rapidly restores systemic perfusion; nonetheless, it increases afterload and may aggravate left ventricular distension, especially in cases of severe LV dysfunction [46]. Adjunctive unloading strategies such as combining VA-ECMO with Impella (ECpella strategy) or IABP may mitigate these effects. Recently, the ECLS-SHOCK trial evaluated early VA-ECMO in patients with AMI-related CS [47]. While there was no significant reduction in 30-day mortality [48], the importance of patient selection, bleeding risk, and the timing of initiation was highlighted. Ongoing trials aim to better define the ideal candidates and protocols for ECMO support.

### 5.3 Device Selection and Strategic Considerations

Each MCS modality had different hemodynamic effects, insertion requirements, and complication profiles.

Device choice should be individualized based on the clinical presentation, institutional expertise, and resource availability. The key considerations include:

-Severity and stage of shock (based on the SCAI classification).

-Degree of left ventricular dysfunction.

-Anticipated duration of support.

-Risk of bleeding, limb ischemia, or hemolysis.

-Logistics of escalation and weaning strategies.

Randomized trials have yet to show a clear mortality benefit for any MCS device; however, timely initiation, especially before irreversible organ damage, appears to improve outcomes in specific patients. A multidisciplinary heart team approach involving cardiologists, intensivists, surgeons, and perfusion specialists is essential to optimize decision-making and care delivery. In addition to hemodynamic parameters and clinical situations, it is essential to consider patient-centered outcomes such as quality of life, functional independence, and long-term rehabilitation potential when deciding whether to escalate or de-escalate MCS. Shared decision-making involving patients and families is particularly relevant in borderline or end-stage cases, where the goals of care may not just focus on survival but also on comfort, dignity, and patient values.

#### Clinical Implications

Managing cardiogenic shock resulting from ACS requires early recognition, close monitoring of hemodynamics, and timely escalation of support. While pharmacological agents remain in the initial stage, their benefits are often transient and must be balanced against the risk of increasing myocardial oxygen demand MCS devices, such as IABP, Impella, and VA-ECMO, should be promptly considered in patients experiencing persistent hypotension or signs of organ hypoperfusion. The choice of device depends on the severity and profile of the shock, institutional experience, and expected duration of support. Notably, delayed initiation of treatment may reduce the impact of this benefit. Device-related complications are significant and require careful patient selection. A structured approach based on the SCAI staging and multidisciplinary input can help align interventions with clinical trajectories and patient goals. In practice, timing and judgment are as crucial as technology.

## 6. Antithrombotic Therapy and Dapt Strategy

Optimal antithrombotic therapy in ACS involves carefully balancing the need to reduce the ischemic risk while preventing bleeding complications. After initiating DAPT, the focus shifted to tailoring treatment duration and intensity based on each patient’s profile, procedural characteristics, and the latest clinical evidence.

### 6.1 Duration and Individualization oF DAPT

In patients undergoing PCI for ACS, the standard recommendation is to administer dual antiplatelet therapy (DAPT) with aspirin and a P2Y_12_ inhibitor for 12 months, provided that the patient does not have an excessive bleeding risk [49]. However, substantial evidence supports a more flexible, patient-centered approach in which treatment duration is tailored according to the individual balance between ischemic and bleeding risks.

To help categorize patients with heightened bleeding vulnerability, the Academic Research Consortium for High Bleeding Risk (ARC-HBR) introduced standardized criteria that define major and minor bleeding risk features [50]. These criteria encompass clinical characteristics commonly associated with serious bleeding events—such as advanced age, a history of major bleeding, severe renal impairment, and the need for long-term oral anticoagulation—as well as less severe but cumulatively important factors, including anemia or chronic NSAID therapy. Incorporating these risk features into clinical decision-making enables a more personalized selection of DAPT intensity and duration, particularly when considering abbreviated or de-escalated regimens in appropriate patients.

Recognizing a patient’s ARC-HBR status is essential, as approximately 20% of patients undergoing PCI meet these criteria. This classification supports personalized antithrombotic strategies, including shortened DAPT duration in selected cases without compromising ischemic protection. Approximately 20% of patients undergoing PCI meet these criteria [50]. In patients with a high bleeding risk (HBR), DAPT duration can be safely reduced. Trials such as STOPDAPT-2 and SMART-DATE have explored early transition to single antiplatelet therapy (SAPT), showing non-inferior ischemic outcomes in low-risk groups and reduced bleeding events [51, 52]. Conversely, extending DAPT beyond 12 months might be beneficial for those at high ischemic risk, such as patients with multivessel disease, DM, or a history of stent thrombosis; nevertheless, at the expense of a higher risk of bleeding. Risk scores such as the PRECISE-DAPT and DAPT scores can help guide duration decisions.

### 6.2 Monotherapy Strategies After Initial DAPT

Recent studies have challenged the conventional sequence of DAPT followed by aspirin monotherapy. Instead, trials have evaluated the efficacy of P2Y_12_ inhibitor monotherapy as a long-term alternative to aspirin, particularly in East Asian populations, where gastrointestinal bleeding is more prevalent. The HOST-EXAM trial compared clopidogrel and aspirin monotherapy following 12 months of uneventful DAPT. It found that clopidogrel significantly reduced the composite of death, MI, stroke, and revascularization without increasing the risk of major bleeding [53]. The SMART-CHOICE 3 trial, which focused on patients with high-risk who had completed standard DAPT, also showed that clopidogrel monotherapy was superior to aspirin in preventing recurrent ischemic events, particularly MI, without an increase in BARC type 2–5 bleeding [54]. These findings suggest that clopidogrel monotherapy may be a viable long-term option for secondary prevention in specific patients, especially those with a HBR or a history of gastrointestinal intolerance to aspirin. P2Y_12_ monotherapy strategies involving ticagrelor have also been extensively evaluated. A meta-analysis conductedby Lee *et al*. [55], which included the TICO, T-PASS, and ULTIMATE-DAPT trials, showed that de-escalation to ticagrelor monotherapy after 3 months of DAPT was associated with a significantly lower risk of major bleeding (0.8% vs. 2.5%; HR, 0.30 [95% CI, 0.21–0.45]) without an increase in ischemic events (1.7% vs. 2.1%; HR, 0.85 [95% CI, 0.63–1.16]). In the TICO trial, Kim *et al*. [56] reported that ticagrelor monotherapy after 3 months of DAPT reduced the composite outcome of major bleeding and adverse cardiac and cerebrovascular events compared with continuing 12-month ticagrelor-based DAPT (HR, 0.66 [95% CI, 0.48–0.92]; *p* = 0.01) [56]. Furthermore, a pooled analysis conducted by Baber *et al*. [57] of the TWILIGHT and TICO trials confirmed that ticagrelor monotherapy significantly reduced major bleeding (0.8% vs. 2.1%; HR, 0.37 [95% CI, 0.24–0.56]; *p* < 0.001) without increasing the risk of death, MI, or stroke (2.4% vs. 2.7%; HR, 0.91 [95% CI, 0.68–1.21]; *p* = 0.515) [57]. Overall, these findings show the importance of tailored monotherapy strategies in contemporary antiplatelet management, providing a promising balance between ischemic protection and bleeding avoidance in selected populations. While SAPT can refer to either aspirin or a P2Y_12_ inhibitor, clopidogrel has become the preferred choice. The HOST-EXAM trial showed that clopidogrel monotherapy 12 months after DAPT was superior to aspirin monotherapy in reducing ischemic and bleeding events. Therefore, clopidogrel is increasingly recommended as post-DAPT monotherapy, especially in East Asian populations.

### 6.3 Triple Therapy and Anticoagulation Considerations

Patients with ACS who require long-term oral anticoagulation, including those with AF, mechanical heart valves, or prior venous thromboembolism, are difficult to manage because of the increased risk of bleeding associated with triple antithrombotic therapy. Historically, these patients received combined aspirin, clopidogrel, and a vitamin K antagonist (VKA). However, modern strategies prefer to reduce the duration of triple therapy. The WOEST trial showed that omitting aspirin from triple therapy significantly reduced bleeding events without increasing the thrombotic risk [42]. Further trials using direct oral anticoagulants (DOACs), including PIONEER AF-PCI, RE-DUAL PCI, AUGUSTUS, and ENTRUST-AF PCI, confirmed the safety of dual therapy (DOAC plus clopidogrel) compared to triple therapy. Current guidelines recommend limiting triple therapy to 1–4 weeks in most patients, followed by at least 6–12 months of dual therapy with a DOAC and clopidogrel, and then transitioning to DOAC monotherapy [58].

Notably, prasugrel and ticagrelor are generally avoided in this setting owing to increased bleeding risk, with clopidogrel remaining the P2Y_12_ inhibitor of choice. Bleeding events are not merely procedural nuisances; however, they are thought to significantly increase morbidity and mortality, with a prognostic impact similar to that of ischemic events. Therefore, personalizing antithrombotic strategies to reduce the risk of bleeding is necessary, particularly in patients receiving triple therapy. Recent data have underscored the clinical relevance of strategies to avoid bleeding in optimizing long-term outcomes in this population [59].

### 6.4 De-Escalation Strategies and Personalized Approaches

De-escalation strategies aim to reduce the intensity and duration of antiplatelet therapy following the acute phase of ACS. This approach balances the need to prevent ischemic events with the risk of bleeding. It may also involve switching from potent P2Y_12_ inhibitors such as prasugrel or ticagrelor to clopidogrel or transitioning from DAPT to SAPT earlier than what standard guidelines recommend. These strategies can be guided or unguided by platelet function testing (PFT) or CYP2C19 genotyping, respectively. In the TROPICAL-ACS trial, a genotype-guided approach using PFT after 1 week of prasugrel showed non-inferiority to continuing prasugrel-based DAPT, with fewer bleeding events [60]. Similarly, the POPular Genetics trial showed that using genotyping to select P2Y_12_ inhibitors allowed safe use of clopidogrel in patients without CYP2C19 loss-of-function alleles, without increasing ischemic risk [61]. These findings support the viability of personalized antiplatelet therapy, particularly in populations with known variability in clopidogrel response.

However, de-escalation should be cautiously considered in high-risk settings. Conditions such as DM, CKD, and chronic total occlusion (CTO) are associated with increased thrombotic risk and stent thrombosis [62]. In such high-risk settings, careful selection and timing of de-escalation strategies are more important, since premature de-escalation may compromise ischemic protection.

Importantly, interpreting these findings requires attention to differences in study design and patient populations. For instance, SMART-CHOICE 3 focused on patients with a high risk of ischemia in East Asia, while TROPICAL-ACS evaluated guided de-escalation in a predominantly European cohort. HOST-EXAM compared aspirin and clopidogrel in stable patients one year after undergoing PCI. Such geographic and demographic differences can affect the bleeding risk profiles, drug metabolism, and adherence patterns. In real-world practice, broader factors such as access to genetic testing, healthcare infrastructure, and patient preferences are also involved. Therefore, the choice of long-term antithrombotic therapy should be guided by a nuanced understanding of evidence and context.

Particularly, CKD has been associated with worse outcomes after ACS, owing to increased platelet reactivity, endothelial dysfunction, and a heightened thrombotic milieu. Moreover, developing acute kidney injury (AKI) during hospitalization for ACS is an independent predictor of mortality and recurrent cardiovascular events. Several large-scale cohort studies have highlighted that even mild or transient AKI confers a long-term risk, highlighting the importance of renal monitoring and early intervention in this population.

#### Clinical Implications

Antithrombotic therapy in ACS warrants balancing thrombotic risk against bleeding vulnerability, an equation that shifts over time and varies among patients. While 12 months of DAPT remains the standard after PCI, recent data support shorter durations in patients with HBR and selective prolongation in those experiencing ischemic risk. The choice and timing of P2Y_12_ inhibitors should account for bleeding profile, need for surgical revascularization, and genetic or functional testing when available. After initial DAPT, P2Y_12_ monotherapy is a reasonable long-term option, particularly in East Asian populations or those with a history of gastrointestinal bleeding. Patients requiring oral anticoagulation therapy should undergo brief triple therapy, and clopidogrel is preferred over more potent agents. Tailoring therapy to patient profiles and periodic re-evaluation may be a better compromise between protection and safety rather than fixed durations or drugs.

## 7. Secondary Preventions After ACS

Long-term management of ACS is more than just revascularization and antithrombotic therapy. Effective secondary prevention strategies focus on reducing the risk of recurrent cardiovascular events, improving survival, and enhancing the patient’s quality of life. Achieving this requires a comprehensive, multidisciplinary approach that combines lifestyle modifications with evidence-based pharmacological treatments.

### 7.1 Lifestyle and Behaviorial Interventions

Smoking cessation is a very powerful intervention for preventing cardiovascular diseases. Even low levels of tobacco exposure can accelerate atherosclerosis, increase thrombogenicity, and hinder endothelial function. Patients should be offered structured cessation programs, pharmacological aids such as nicotine replacement therapy or varenicline, and ongoing counseling. Electronic cigarettes are not a safe alternative as they may increase sympathetic tone and oxidative stress [63]. Physical activity and dietary modification are essential. Patient’s engagement in moderate-intensity aerobic exercise for at least 150 min/week should be encouraged. The Mediterranean diet, rich in fruits, vegetables, whole grains, fish, and healthy fats, reduces cardiovascular risk. Weight reduction is recommended for patients with obesity (body mass index ≥25 kg/m^2^ in Asian populations), with a target of 5–10% reduction in body weight over 6–12 months [64]. Psychosocial factors, including depression, social isolation, and work-related stress, are independently associated with worse cardiovascular outcomes. Screening and appropriate management of these factors, as well as referral to cardiac rehabilitation programs, are essential.

### 7.2 Lipid Management

Reducing the low-density lipoprotein cholesterol (LDL-C) levels is the key to secondary prevention. Current guidelines recommend an LDL-C target of <55 mg/dL (1.4 mmol/L) and at least 50% reduction from baseline in patients with very high risk, including those with ACS [13]. Ideally, high-intensity statin therapy should commence as early as possible during hospitalization. If the aim of LDL-C is not achieved despite maximally tolerated statin therapy, ezetimibe should be administered. In cases of persistent elevation, especially in patients with familial hypercholesterolemia or DM, pro-protein convertase subtilisin/kexin type 9 (PCSK9) inhibitors should be used. These agents effectively reduce LDL-C levels and show favorable effects on plaque stabilization and regression. The 2023 ESC Guidelines recommend reassessing lipid levels 4–6 weeks after initiating or modifying treatment, with subsequent titration as needed. PCSK9 inhibitors offer potent lipid-lowering effects; nevertheless, their high cost has raised concerns regarding cost-effectiveness [65]. Consequently, it is advisable to carefully select patients, such as those with a very high residual risk, especially in countries with constrained healthcare resources.

### 7.3 Hypertension and Blood Pressure Control

Hypertension significantly contributes to the recurrence of cardiovascular events. The general target for blood pressure is <140/90 mmHg, while more aggressive targets (<130/80 mmHg) are considered in patients with DM, CKD, or a high risk of stroke [13]. For patients with left ventricular systolic dysfunction, anterior MI, DM, or CKD, angiotensin-converting enzyme (ACE) inhibitors or angiotensin receptor blockers (ARBs) are recommended. Beta-blockers are beneficial for reducing myocardial oxygen demand, especially in patients with reduced ejection fraction or those at risk of arrhythmias. Calcium channel blockers may be an option for patients experiencing persistent angina or intolerance to first-line agents.

### 7.4 DM and Glycemic Control

M significantly increases the risk of adverse outcomes following ACS. The general target for hemoglobin A1c (HbA1c) is <7%; however, individualized goals are appropriate for older or frail patients. Recently, antidiabetic agents with proven cardiovascular benefits have changed the therapeutic paradigm. Among these, glucagon-like peptide-1 receptor agonists (GLP-1 RAs), such as liraglutide, semaglutide, and dulaglutide, have shown significant reductions in MACE across several large cardiovascular outcome trials (CVOTs). The LEADER trial reported a 13% relative risk reduction in MACE with liraglutide, whereas the SUSTAIN-6 trial showed a 24% reduction with semaglutide [66].

These agents enhance glycemic control and provide additional benefits, including weight loss, reduction in systolic blood pressure, and anti-inflammatory effects. Together, these advantages contribute to cardiovascular protection. In recognition of this evidence, the ESC and American Diabetes Association now recommend GLP-1 RAs with proven cardiovascular benefits for patients with type 2 diabetes mellitus (T2DM) who have established atherosclerotic cardiovascular disease or high cardiovascular risk [67].

These guidelines are based on strong and consistent findings from CVOTs, confirming the safety and efficacy of these agents in reducing cardiovascular morbidity and mortality. While GLP-1 RAs have not been extensively studied in the immediate post-ACS period, using them during the early outpatient phase appears to be safe and advantageous, especially in patients with poor glycemic control, obesity, or multiple cardiometabolic risk factors [68]. Further studies are needed to establish optimal timing for the post-ACS treatment continuum. DM significantly increases the risk of adverse outcomes after ACS. HbA1c targets should generally be <7%; however, individualized goals are appropriate for older or frail patients. Sodium-glucose cotransporter 2 (SGLT2) inhibitors and glucagon-like peptide-1 (GLP-1) receptor agonists are key agents in patients with T2DM and established atherosclerotic cardiovascular diseases. These drugs improve glycemic control and reduce cardiovascular events and hospitalization due to heart failure. Initiating these agents should be considered early in the post-ACS period, especially in patients with poor glycemic control or multiple comorbidities [68].

### 7.5 Anti-Inflammatory Therapies

Inflammation is key to destabilizing atherosclerotic plaques. Conventional therapies, such as statins, have modest anti-inflammatory effects. However, targeted anti-inflammatory agents are being actively explored. Colchicine, an inexpensive and well-tolerated drug, has reduced cardiovascular events in recent trials such as COLCOT and LoDoCo2 [69, 70]. In the COLCOT trial, low-dose colchicine (0.5 mg/day) administered within 30 days of MI significantly reduced the composite endpoint of cardiovascular death, MI, stroke, and urgent revascularization. These results support its consideration as an adjunctive therapy in selected patients; however, it is important to note that while colchicine reduces cardiovascular events, it does not significantly impact all-cause mortality or cardiovascular mortality [71]. Some studies have reported an increase in noncardiovascular deaths, warranting careful patient selection and monitoring.

### 7.6 Antithrombotic Strategy Beyond One Year

After completing the standard 12-month DAPT regimen after ACS, the long-term antithrombotic strategy must be tailored following the balance between ischemic and bleeding risks. Aspirin has traditionally been the default SAPT; however, this convention has come under scrutiny recently. The SMART-CHOICE 3 trial provided compelling evidence to support the use of clopidogrel monotherapy in patients with high ischemic risk who completed a standard DAPT course after undergoing PCI [54]. In this large-scale randomized study, patients were assigned to receive long-term maintenance therapy with either clopidogrel or aspirin monotherapy. Clopidogrel significantly reduced major cardiovascular events, including myocardial infarction, and showed a favorable safety profile with fewer gastrointestinal adverse events and no increase in major bleeding. These findings marked the first evidence of clopidogrel’s superiority over aspirin in a broad post-PCI population. This challenged the default role of aspirin for long-term use and suggested that clopidogrel may be a better choice in patients with aspirin intolerance, a history of gastrointestinal bleeding, or heightened risk of bleeding, especially in East Asian populations where such risks are elevated. For patients with a persistently high ischemic risk and low risk of bleeding, extended DAPT remains an option. The PEGASUS-TIMI 54 trial showed that continuing DAPT with low-dose ticagrelor (60 mg twice daily) alongside aspirin significantly reduced the risk of myocardial infarction and stroke in patients with a history of MI, albeit at the cost of a modest increase in bleeding [72]. This regimen may be appropriate in patients with multivessel disease, complex PCI, or a history of stent thrombosis. Additionally, the COMPASS trial showed that a low dose of rivaroxaban (2.5 mg twice daily) combined with aspirin significantly reduced cardiovascular events in patients with stable atherosclerotic disease. This regimen may be considered for selected high-risk patients with low bleeding risk after a year. However, the generalizability of these findings warrants careful consideration. Trials such as SMART-CHOICE 3 and STOPDAPT-2 were primarily conducted in East Asian populations, which have different pharmacodynamic and bleeding risk profiles compared to Western populations. This so-called “East Asian paradox”—characterized by a lower ischemic risk but higher bleeding susceptibility—may affect the safety and efficacy of long-term antithrombotic strategies. Consequently, while clopidogrel monotherapy appears to be particularly beneficial in East Asian patients, extrapolation of these results to broader global populations should be made cautiously, ideally supported by additional trials in more diverse cohorts.

### 7.7 Long-Term Adherence and Systems of Care

Ensuring adherence to evidence-based therapies is vital. Suboptimal adherence is linked to increased risk of infarction and mortality. To enhance long-term compliance, simplifying medication regimens, involving pharmacists and nurses in medication counseling, and utilizing reminder systems or mobile health applications can be effective. Cardiac rehabilitation programs that combine supervised exercise, education, nutritional counseling, and psychological support have been shown to lower mortality and hospital readmissions. Therefore, participation in the study was encouraged soon after being discharged. In aging societies such as Japan, it is essential to consider polypharmacy, drug tolerability, and patient preferences. Shared decision-making involving caregivers and multidisciplinary teams is becoming increasingly important for optimizing secondary prevention in complex cases.

Effective secondary prevention after ACS requires pharmacological optimization and lifestyle intervention modifications as well as long-term adherence strategies tailored to the patient’s comorbidities and preferences. Randomized trials, such as TWILIGHT, TROPICAL-ACS, and SMART-CHOICE, have shaped contemporary de-escalation strategies; nevertheless, they often exclude high-risk groups and are limited by their open-label designs and relatively short follow-up periods. Therefore, clinicians should interpret these findings considering the complexities of real-world scenarios. Complementary evidence from meta-analyses and observational registries can enhance external validity, but should be distinguished from randomized data in terms of strength and generalizability.

#### Clinical Implications

Secondary prevention after ACS goes beyond pharmacology and depends on sustained patient engagement. Lipid-lowering therapy, blood pressure control, and glycemic management remain central; however, they require ongoing reassessment and titration. GLP-1 receptor agonists and SGLT2 inhibitors provide cardiometabolic benefits beyond lowering glucose and should be considered early in appropriate patients. Smoking cessation, dietary change, and structured exercise have a clear prognostic value; however, they are often underused. Addressing psychosocial stress, depression, and medication adherence is essential, especially in aging or multimorbid populations. Cardiac rehabilitation is key to reinforcing these elements. The most effective approaches often combine medical therapy with practical support, individualized follow-up, and shared decision-making, all tailored to align with the patient’s self-care goals and capacity.

## 8. Conclusion

Recent advances in diagnostic algorithms, imaging techniques, antithrombotic strategies, and secondary prevention have enabled a more personalized approach to managing ACS. High-sensitivity troponin assays and multimodal imaging enable early and accurate diagnoses, whereas tailored revascularization and de-escalated antithrombotic therapy enhance patient outcomes and reduce adverse events. As clinical evidence grows, guidelines are evolving to reflect precision-based care. However, certain gaps remain, particularly regarding the routine use of intracoronary imaging and mechanical support devices. Future research should focus on refining strategies that align with patient-specific profiles, values, and long-term functional goals. A truly personalized ACS care model requires the integration of clinical, biological, and patient-reported factors into shared decision-making.

## Abbreviations

ACS, acute coronary syndrome; ECG, electrocardiogram; ED, emergency department; AMI, acute myocardial infarction; hs-cTn, high-sensitivity cardiac troponin; CKD, chronic kidney disease; DM, diabetes mellitus; NSTE-ACS, non-ST-segment elevation acute coronary syndrome; DAPT, dual antiplatelet therapy.
